# Choice of the Dialysis Modality: Practical Considerations

**DOI:** 10.3390/jcm12093328

**Published:** 2023-05-07

**Authors:** Massimo Torreggiani, Giorgina Barbara Piccoli, Maria Rita Moio, Ferruccio Conte, Lorenza Magagnoli, Paola Ciceri, Mario Cozzolino

**Affiliations:** 1 Néphrologie et Dialyse, Centre Hospitalier Le Mans, 194 Avenue Rubillard, 72037 Le Mans, France; maxtorreggiani@hotmail.com (M.T.); mariaritamoio@gmail.com (M.R.M.); 2 Renal Division, Department of Health Sciences, Uiniversity of Milan, San Paolo Hospital, 20142 Milan, Italy; conteferruccio47@gmail.com (F.C.); lorenza.magagnoli@unimi.it (L.M.); p.ciceri@hotmail.it (P.C.); mario.cozzolino@unimi.it (M.C.)

**Keywords:** chronic kidney disease (CKD), kidney replacement therapy (KRT), conventional hemodialysis, home hemodialysis, peritoneal dialysis, predialysis education, incremental dialysis

## Abstract

Chronic kidney disease and the need for kidney replacement therapy have increased dramatically in recent decades. Forecasts for the coming years predict an even greater increase, especially in low- and middle-income countries, due to the rise in metabolic and cardiovascular diseases and the aging population. Access to kidney replacement treatments may not be available to all patients, making it especially strategic to set up therapy programs that can ensure the best possible treatment for the greatest number of patients. The choice of the “ideal” kidney replacement therapy often conflicts with medical availability and the patient’s tolerance. This paper discusses the pros and cons of various kidney replacement therapy options and their real-world applicability limits.

## 1. The Growth of the End-Stage Kidney Disease Population

Chronic kidney disease (CKD) affects about 10% of the world population and, in high-income countries, where kidney replacement therapy is available without restrictions, over one individual over 1000 lives thanks to dialysis or kidney transplantation; while the prevalence of CKD is currently estimated to be over 10% in high and low-income countries, the access to dialysis and transplantation is uneven, and these life-saving and life-sustaining treatments are still available for less than one-third of the world population [1,2,3,4].

**Figure 1:**
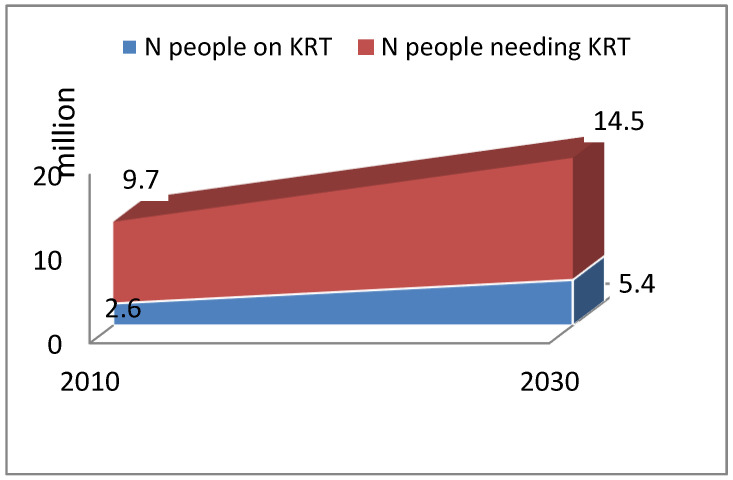
Worldwide predicted estimation of the need for and the possibility of access to kidney replacement therapy (Adapted from [4]).

Despite the limitations, the prevalence of patients living on kidney replacement therapy (KRT) has increased greatly during the last decades [1,2]. While in high-income countries CKD and End-Stage Kidney Disease (ESKD) maintain a rather constant incidence over the last decades, low- and medium-income countries are experiencing a dramatic increase in CKD and ESKD [3,4,5]. It has thus been estimated that the current population of 2.5–3 million people treated with KRT will double in the next decade if the financial resources will allow it. Lack of access to KRT would, on the contrary, increase deaths from kidney failure (Figure 1) [4,5]. The discrepancy between the need for KRT and the availability of dialysis and transplantation has already been pointed out on several occasions [6]. Several factors modulate the expected worldwide increase in patients requiring KRT: decrease in competitive mortality, leading to increased life expectancy in the general population but also to a greater load of comorbidities such as diabetes, cardiovascular diseases, and hypertension; growth in the prevalence of CKD due to the aging of the population and the effect of the comorbidities; increased access to dialysis in low- and middle-income countries; reduction of mortality in KRT patients, as a result of technical and medical advances in high-income countries and to the improvement and increased availability of care in medium-low-income countries [4,5,6]. Treatment choices for patients with ESKD are many, ranging from pre-emptive kidney transplantation to conservative therapy. Where several options are available, the choice of KRT requires a shared decision with the patients and their families, aiming not only to ensure the best survival but also to preserve and improve the quality of life.

## 2. The “Ideal” Treatment

Kidney transplantation, when possible and available, is currently the best treatment for ESKD patients without contraindications [7,8]. In these patients, pre-emptive transplantation is even superior, ensuring better organ and patient survival [7]. However, pre-emptive transplantation is not always possible, due to the limited availability of living and deceased donors, and the lack of healthcare structures. Furthermore, the attitude towards donation and access to kidney transplantation is highly variable, depending on the cultural background, and is influenced by ethnicity, educational level, access to the health care system, setting of care, logistic issues (for example distance from the centers of care) and comorbidity [7,8,9]. Each year, new patients are wait-listed for transplantation and a similar number leaves the waiting list not only because of transplantation but sadly, in about 25% of the cases, due to death or deterioration of health [10]. In the last decades, due to the progressive improvement in immunosuppressive treatments and, to a lesser extent, in surgical techniques, kidney transplantation became an option also for patients with severe comorbidities and the age limit has been progressively increased up to 80 years, and, occasionally, even beyond. Notwithstanding these improvements, only between 30% and 70% of patients with ESKD are good candidates for kidney transplantation [8,9,10]. Considering the limited availability, the most widely used treatments for KRT are the different dialysis techniques (Table 1), usually categorized according to the extracorporeal (“hemodialysis”, HD) or intracorporeal (Peritoneal dialysis, PD) approach. Dialysis treatments can also be categorized by the timing of prescription: urgent vs. non-urgent, programmed vs. unprogrammed, and the relative combinations.


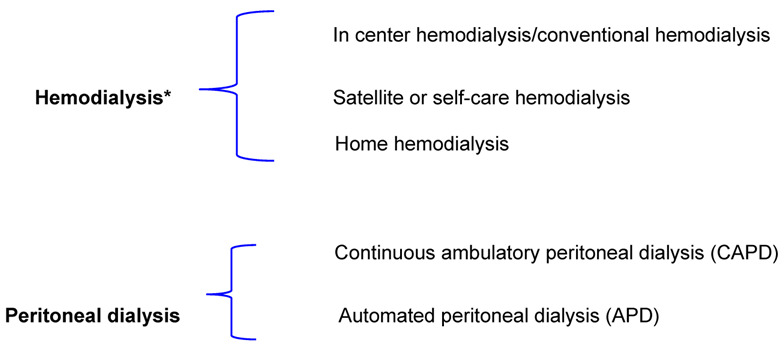


* with the term hemodialysis we indicate here all the extracorporeal techniques, namely hemodialysis and the different forms of hemodiafiltration (hemodiafiltration, acetate-free biofiltration, etc.).

## 3. The Best Treatment: The Nephrologist’s Point of View

The term “dialysis adequacy” has been classically used to identify a treatment “at target” according to the kinetic of urea, chosen for its simplicity, low cost, and easy and repeatable calculation. On hemodialysis, the urea KT/V, and on peritoneal dialysis the weekly Kt/V and the PET are indexes based on small solute clearance, considered to be precise enough to target efficiency without the need for a more comprehensive assessment. However, the exclusive use of these important markers of depuration efficiency limits the primary objective of dialysis on a single parameter and hinders the implementation of multidimensional approaches, in terms of clinical issues (control of anemia, nutritional status, bone disease, for example) or patient preferences.

The recent and revolutionary 2020 guidelines for the management of “high-quality” peritoneal dialysis put the quality of life first, and small-molecule clearance appears only at the bottom of a long list of issues to be considered for a successful dialysis prescription [11]. While guidelines for the management of hemodialysis still set “efficiency first”, the increased use of incremental and personalized schedules is likely to shift the focus on patients’ perceptions and quality of life.

Indeed, the relevant issues for dialysis modulation may vary according to the physician’s and patient’s points of view. For instance, in a recent Delphi survey, aiming to identify outcomes to be used in hemodialysis trials, some common goals were prioritized for both groups (vascular access, dialysis adequacy, fatigue, cardiovascular disease, and mortality), but patients/caregivers privileged lifestyle-related outcomes such as the ability to travel, dialysis-free time, dialysis adequacy, and not being “washed out” after dialysis [12]. Several recent studies underline how patient satisfaction should be included among dialysis outcomes to be measured in trials as well as considered in dialysis choice [12,13,14,15,16,17,18].

According to the EPOCH-RRT study, the inclusion of patients’ priorities in the choice of dialysis treatment improves outcomes [19]. The complex issues of dialysis initiation, modality choice, dialysis dose prescription, and choice of vascular access were addressed by a recent KDIGO “Controversies Conference” [20]. The main message coming from the conference is that a planned start of dialysis should be decided through a shared choice with patients and caregivers, evaluating not only the best program but its acceptability and intrusiveness in daily life [20]. However, treatment modalities are often modulated by other limiting factors. Overcoming limitations is difficult and often not affordable, but identifying the barriers may draw the attention of health authorities towards overcoming obstacles and shared choice of kidney replacement treatment, respecting the quality of patients’ daily life [20].

## 4. Predialysis Education

In the choice of the dialysis modality, the decision-making process shared between doctor and patient should be started in advance to limit the patient’s anxiety in facing a radical lifestyle change [21]. The moment of choice is difficult, and there are no standardized eGFR values at which to start education and preparation for dialysis, as the choice should depend on the individual kidney function trajectory, social context, and personal preferences. In the protocol of the “GUIDE” study, the authors standardized the eGFR level of 15 mL/min/1.73 m^2^ (20 mL/min/1.73 m^2^ in patients with rapid deterioration of renal function) as the time to start education and preparation for KRT [22]. However, this may be too late for some patients or too early for others. An educational pathway, whenever possible with the availability of multidimensional supports, is probably the best way to reach a shared decision on the type and timing of dialysis initiation and to ensure patient empowerment, which is now identified as an important safeguard for a reduction in mortality, specifically at dialysis start [23].

A pivotal study conducted on over three thousand attendees in the Fresenius Kabi Centers in the US (10.5% of 30,217 incident patients admitted between 1 January and 31 December 2008), demonstrated that even accounting for the selection of the most collaborative and empowered patients, attending a pre-dialysis option class was associated with more frequent selection of home dialysis, fewer tunneled HD catheters, and lower mortality risk during the first 90 days of dialysis therapy [24].

A recent observational study on 1117 patients starting dialysis in the same center over 10 years showed that a pre-dialysis period of 20 months significantly reduced the risk of death in the first year (HR 0.58: *p* = 0.040) [25].

In a recent French study assessing the results of a systematic start of incremental hemodialysis, in the absence of contraindications, patients followed up in an “intensive” pre-dialysis clinic had 10 times higher odds of having a smooth, incremental start of hemodialysis [26].

The first months of dialysis are crucial for patient survival, with a mortality peak of around 90–120 days, then decreasing and getting more constant from 12 months onwards. This excess mortality is more evident in hemodialysis than in peritoneal dialysis, possibly because peritoneal dialysis is less frequently started in an emergency, without a previous follow-up [25]. Data on early mortality in patients starting with incremental hemodialysis are needed to better understand what the role of a gradual start of KRT is, which is routinely practiced in peritoneal dialysis and only exceptionally on hemodialysis [27,28].

## 5. Dialysis Modalities

**Figure 2:**
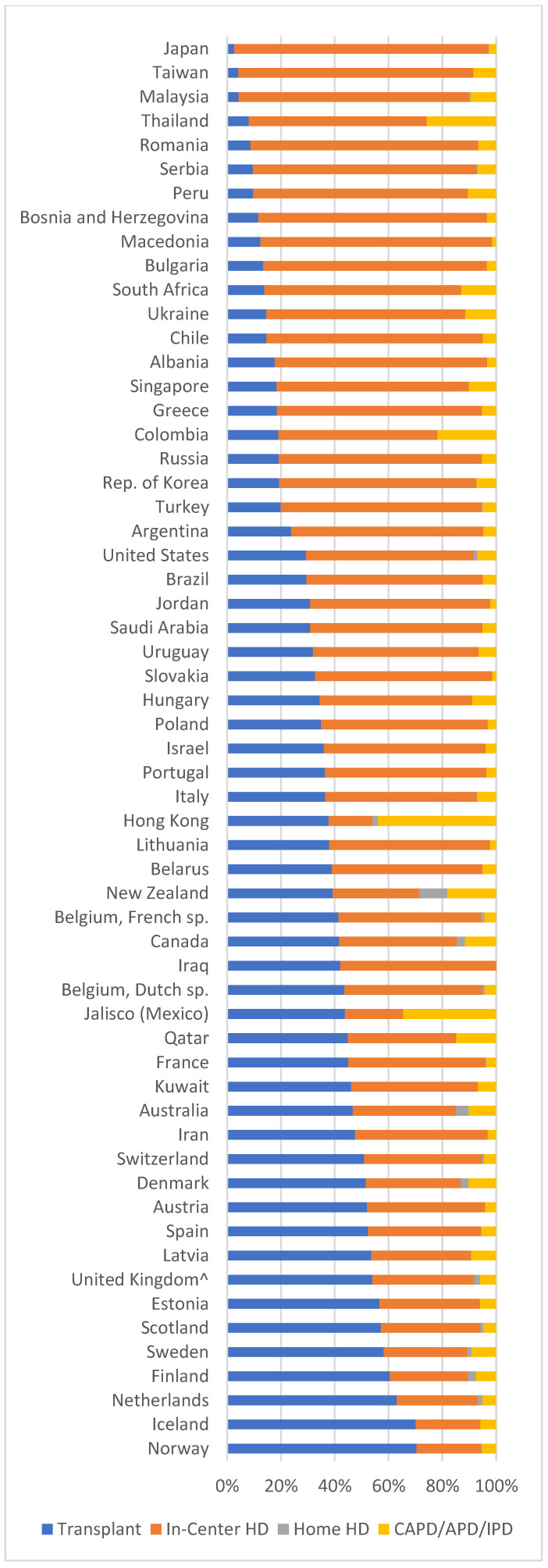
ESRD treatment modalities worldwide (Adapted from the United States Renal Data System. 2018 USRDS Annual Data Report: Epidemiology of kidney disease in the United States. National Institutes of Health, National Institute of Diabetes, Digestive, and Kidney Diseases, Bethesda, MD, 2018).

The distribution of the dialysis modalities is uneven, and the differences across countries are remarkable, showing the importance of the social, cultural, and economic setting [1,4,5]. According to the United States Renal Data System (USRDS) registry, in 2017 62.9%, of prevalent patients were treated with HD, 7.1% with PD, and 30% with kidney transplantation [29]. In a recent survey by the Italian Society of Nephrology, out of 60,441 patients on KRT, 51% were on HD, 7% on PD, and 42% profited from kidney transplantation [30]. Some data on the distribution of ESKD treatment modalities worldwide is provided in Figure 2. Worldwide, 89% of dialysis patients living in high–medium-income countries are on HD, and the use of this modality is growing faster in Latin-American countries than in Europe and the USA. The dialysis treatment more extensively, and sometimes exclusively, available is in-hospital hemodialysis.

The distribution of home dialysis, in both forms of home hemodialysis (HHD) and peritoneal dialysis (PD) varies widely, being the highest in Hong Kong, Mexico, Canada, and Australia-New Zealand, and varying also within European countries, from the highest values in Finland and Norway to the lowest in Mediterranean ones [1,31].

This review will not discuss the issue of indications and contraindications to kidney transplantation but will focus on the type of dialysis treatment and its settings.

### 5.1. Home Dialysis Treatments

Home dialysis first is an old, but still valuable, paradigm coined in the late eighties by Oreopulos, one of the fathers of peritoneal dialysis [32]. Home-based treatments merge flexibility, usually, deliver a higher dialysis dose, require and value patient participation, and what is now called “patient activation”, which is associated with a higher quality of life and better survival [33,34,35]. While the endless discussion on whether the good results obtained in home hemodialysis and peritoneal dialysis are linked to patient selection, since only the most motivated and compliant patients chose home-based treatments, or to a favorable case mix is still open, it is clear that a home dialysis choice is associated with good results for the patient and with a lower burden for the society [36]. Hence, home treatments should be encouraged, and different from the present situation, the reimbursement fees should reward these choices.

The recent pandemic clearly showed the further, unexpected, advantage of limiting exposure to the hospital setting, as patients on home dialysis were significantly spared from contaminations, which resulted in lower mortality in the first phases of the pandemic before vaccines became available [37,38]. The choice of a home dialysis modality cannot be shared with the patient. The modality of reaching this shared choice is very culturally sensitive [39,40]. The importance of a well-organized program focusing on the improvement in freedom, flexibility, well-being, and strengthening of relationships, has been clearly shown, among others, by the study “GUIDE” [22]. Choosing a home dialysis treatment requires overcoming several barriers for patients, families, and sometimes institutions. Some of these are listed in Table 2, while the main benefits are summarized in Table 3, and Table 4 shows the contraindications to home treatments. We have to acknowledge, however, that experiences on home dialysis are not always positive and, when home dialysis was the only way to survive, there were experiences of being ”trapped in the disease”. Thus, an important point is the availability of an easy transfer from home to in-center care, considering dialysis not as a single therapy option but as an integrated system of care offering different modalities and settings. A dialysis modality, chosen at the beginning of treatment, sometimes in unplanned situations, may no longer be adequate over time, due to a variety of factors, including changes in the lifestyle of the patient and the family, different needs, medical indications, or unexpected complications [41,42]. On the other hand, home dialysis involves family members and/or caregivers who became valuable allies in providing adequate patient care. Involvement in patient care can be rewarding, but this workload can overburden caregivers as well as patients, inducing depression, frustration, and in some cases, even financial burdens [43,44]. These situations, if protracted and not promptly solved, may lead to failure of home dialysis, with an unfavorable return to in-center care, as reported by the Australian and New Zealand Registry where home dialysis failure is associated with a significant increase in mortality [45].

A constant barrier to home dialysis therapies is the lack of dedicated and supported implementation programs. In this regard, in July 2019, the US administration signed an executive order to promote home dialysis as the main treatment for all patients with end-stage kidney failure, with the ambitious aim of 80% of patients starting KRT on home dialysis or preemptive transplantation [46]. While the authors of this paper doubt the feasibility of such a program, they are aware of the importance of this message, since the feasibility of home dialysis depends also upon political engagement: allowing patients to be empowered implies as well an increase in time invested in care [47,48].

### 5.2. Peritoneal Dialysis

Although PD has been used since the 1960s as an intermittent dialysis treatment, it was not until 1975, when Moncrief and Popovich proposed the concept of continuous dialysis, later defined as Continuous Ambulatory Peritoneal Dialysis (CAPD), that PD began a viable long-term treatment for ESKD [49]. This treatment modality is not the most prevalent one around the world [50,51]. In fact, according to a recent report, only about 11% of dialysis patients worldwide are treated with PD [51]. However, the trend of moving dialysis closer to patients’ homes supports PD (both CAPD and Automated Peritoneal Dialysis -APD-) as the first choice for KRT. Indeed, PD is the most popular home dialysis modality worldwide. Multiple advantages of starting KRT with PD have been reported (Table 3), the most relevant of which is the preservation of residual kidney function and venous capital [21]. Furthermore, some authors demonstrated that pre-transplant PD is associated with better post-transplant survival and a reduced risk of delayed graft function compared to HD, with no difference in graft survival [52]. Thus, PD appears to be the ideal bridge to transplantation [53,54]. Moreover, at least in some settings, the quality of life (QoL) seems to be better on PD, with a lower impact on daily life, easier maintenance of independence, and flexibility in the dialysis schedule. These findings are however not unambiguous in the literature: indeed, some reports show the superiority of QoL with PD, others equivalence, and others inferiority [55,56,57]

Discordant data also regard mortality, once again mainly because of differences in the case mix. Data from the USRDS 2020 Annual Data Report, analyzing mortality in the first 12 months after the start of KRT, after adjustment for the main demographic variables, demonstrate reduced mortality in PD patients compared with HD patients. This difference in mortality is particularly evident in the first 3 months after the start of treatment [31]. However, when the comparison is performed in patients potentially eligible for both methods, no advantage in early mortality is found [58].

As a consequence, the international guidelines recommending PD as the first dialysis modality should be implemented with caution to avoid a high number of dropouts. According to a study by Pulliam and co-workers, in 1677 incident PD patients in the USA, transfer to HD at 6 months was 20.9%, whereas in a similar French study transfer to HD was only 6.3% in the 1st year, although the penetrance of PD in France is low, just over 7 percent [59,60,61]. Both studies showed that patients who needed to discontinue PD had higher morbidity and mortality in HD [59,60]. According to the Australian and New Zealand Registry, the most important risk factors for early dropout from PD were: age > 70, BMI < 18.5 kg/m^2^, diabetes mellitus, ischemic heart disease, peripheral vascular disease, transfer from another dialysis modality, late referral, and being treated in a small center [62]. Although PD is, almost by definition, a self-managed treatment, the association of comorbidities and frailty with advancing age led to the implementation of assisted PD in several settings [63]. This option is differently used in different countries, employing health care workers, public or private community nurses, or relying on family caregivers. Assisted PD (either CAPD or APD) allows for treating at-home patients who, otherwise, would have to travel back and forth to the dialysis center; moreover, the cost balance is usually favorable compared to in-center HD [64,65]. Different combinations of dialysis modalities are available in different countries; for example, about 20% of peritoneal dialysis patients in Japan are treated in combination with weekly hemodialysis. The main reason for this combination is related to the progressive loss of ultrafiltration capacity of the peritoneum, which is balanced by the fluid loss achieved by means of the hemodialysis treatment. A treatment made of 6 days of PD + 1 hemodialysis was shown to be valuable in reducing all-cause and cardiovascular mortality and heart failure [66].

### 5.3. Home Hemodialysis (HHD)

Only a minority of patients begins KRT with HHD. Although HHD has been associated with several clinical benefits, including blood pressure control, and phosphate control, as well as lower use of medications, HHD remains under-represented [67]. Patient selection, enrolment, and training represent the critical step. While the technical training for home PD is short (generally ≤7 days), the training for HHD may be longer and more demanding. Setting up an HHD program requires a “critical mass” of patients and relevant technological and human resources, often available only in large centers. In a study in which the cost of setting up an efficient referral center was analyzed, financial neutrality was achieved only after about 122.6 months [68]. Once more, careful patient selection is important. In analogy with PD, early drop-out from HHD affects survival. The most suitable individuals are those who are motivated to continue working or studying and able to self-manage care; the proposed home hemodialysis treatment should be thoroughly explained, if possible early in the pre-dialysis course, to empower patients [67]. One-third of patients with HHD manage their treatment independently without a partner, while the availability of a trained caregiver expands the applicability of HHD [69].

The different case mix, the higher empowerment, and the fact that HHD patients often dialyze more than prescribed are the main reasons that explain an overall better survival on HHD. Another non-secondary advantage is the possibility of adopting an intensive and personalized dialysis schedule. This flexibility in defining frequency and duration to match the patient’s activities allows for optimizing the control of hypertension, extracellular volume, and hyperphosphatemia while reducing the use of drugs [70]. The most common feasible schemes, each with different advantages, are short daily dialysis, alternate-night dialysis, low-flow daily dialysis, and low-flow night dialysis. The statistically significant variables emerging from published papers associated with choosing HHD are: younger age, a higher educational degree, higher income, longer life expectancy, home ownership, responsibility for child care, and extensive travel options [39,71].

Two large registries (USRDS and ANZDATA) compared mortality and technique failure in incident PD and HHD patients [72,73]. In the 10710 patients in the ANZDATA registry, HHD was associated with a lower risk of death (HR: 0.47: 95%CI, 0.38–0.59) and technique dropout (HR, 0.34; 95% CI 0.29–0.40). These findings were confirmed after adjustment for age and diabetes. While the quest for the best dialysis continues, the emphasis on patient-oriented, shared choices is increasingly shifting towards the identification of the treatment most suited to each individual, and, in this context, HHD is probably the best combination of feasibility, acceptability, and good results for those who select it.

## 6. The COVID-19 Epidemics and Home Treatments

An almost unexpected advantage of home-based treatments, and, to a lesser extent, of dialyzing in smaller settings, has been revealed during the COVID-19 epidemic. Patients less exposed to the hospital milieu had lower chances to die, in particular during the first waves of the epidemics, when vaccines were not yet available. While mortality remained high in infected patients, being mainly driven by the comorbidity burden, the lower exposure led to lower mortality in home dialysis patients, and the need for limiting contact with the hospitals led to the rapid development of remote monitoring, a lesson that can be capitalized for the future [30,74,75,76,77,78,79,80,81].

In fact, in a large study from the US, patients on HHD had between 30 and 50% lower odds of being infected by SARS-CoV-2 and between 30 and 60% lower odds of being hospitalized for COVID-19 compared to in-center hemodialysis patients throughout the first year of the pandemics [80].

Remote monitoring techniques developed for PD patients could easily be adapted to HHD ones. Indeed, in Italy, one of the earliest touched countries, it was reported that in the first two months of the pandemic, a remote monitoring approach resulted in only one infection out of 36 PD patients [81]. It is worth mentioning that the old age of the patient and his habitual reluctance to technology were not a barrier to setting up the remote monitoring, suggesting the large feasibility of this approach.

## 7. Hybrid Choices: Limited Care, Self-Care, Satellite Dialysis

Some “hybrid” solutions try to merge the advantages of self-care without the constraints of having a family partner or a suitable home; these have many names (self-care, limited-care, satellite) which underline the fact that different solutions are proposed in different places. The classical definitions are the following: a self-care dialysis facility is a dialysis facility where the patient performs their dialysis treatment with little or no professional assistance [82]; a limited care dialysis facility is a dialysis facility where there is specialized paramedical personnel to help clinically stable patients performing their treatment in a non-hospital facility [83]; a satellite dialysis facility is a dialysis facility where maintenance hemodialysis is provided in a hospital setting to adult patients with ESKD but these patients are supposed to be stable and do not need the highly specialized treatment provided in the main renal center [84]. The original idea was to offer a setting for the care of patients fit to perform home hemodialysis, but with unsuitable homes or without a dialysis partner [85,86,87]. The further evolution, at least in some settings, such as Italy, and to a lesser extent, in France, tended to favor treatment in proximity, and the treated population shifted from young, completely autonomous patients, to elderly patients in which the discriminating issue for the choice is not the full autonomy but the “low-risk” of intradialytic complications [61,88,89,90].

Likewise, the Canadian experience of treating patients with ESRD in satellite units starting in the mid-1990s had the goal of bringing dialysis closer to patients’ homes, especially for those unable to perform home hemodialysis [91]. Patient care in satellite units is provided by nephrologists working in referral centers, who, besides regular visits to the unit, are in contact with the nurses via telephone, fax, and telehealth [92]. Due to the almost ubiquitous increase in age and comorbidity, the presence of the physician in these units is increasing, with various solutions, including the displacement of a part of the outpatient activity. While in some settings, such as Italy, different dialysis modalities are available in the satellite centers (including hemodiafiltration), in others, such as France, only hemodialysis is available, and this can be a limitation in the case of patients needing a more performing technique for the depuration of the middle molecules [61]. These limitations are counterbalanced by the higher patient empowerment (associated with better outcomes), and by the fact that patients usually dialyze in a more “home-like” and less “hospital-like” setting, which may also lead to better outcomes [93]. Despite the changing case mix of dialysis patients, the definitions of the different dialysis facilities remain the same for the legislator and in terms of reimbursement. Nowadays, to cope with the growing population of patients needing KRT, satellite units often display a selection of pluricomorbid patients never seen before. Moreover, the possibility of kidney transplant beyond 80 years of age has negatively selected even more the dialysis population.

## 8. Conventional Hemodialysis

In-hospital hemodialysis is currently the most widespread dialysis modality (Table 5). This is mainly due to the increase in patients’ age and comorbidity burden [94,95,96].

A recent prospective study carried out in 15 nephrology departments, supplying both PD and HD, in seven Northern European and Baltic states, analyzed the choice of dialysis modality of 1587 consecutive incident ESKD patients [97]. Of these, 32.5% were not able to perform a choice for clinical reasons or because of urgent dialysis need, while of the remaining ones 61.7%, chose PD, 3.6% HHD, and 34.6% in-center HD. Therefore, only 23.4% of patients who were able to select their KRT chose in-center hemodialysis, even though 55.9% started KRT in-center [97]. This study once more quantifies how KRT choice is affected by late referral or urgent dialysis start. Table 6 shows some of the most common barriers to the correct dialysis modality choice.

The importance of education is underlined by a recent survey of patients’ perceptions of factors influencing their choice, involving 7820 patients from 38 countries [98]. Only 25% of in-center HD patients stated that they had not received information concerning the choice of other dialysis modalities and only 23% received information > 12 months before KRT initiation. Patients were not informed about home hemodialysis (HHD) (42%) and comprehensive conservative management (33%) [99]. Information regarding available dialysis modalities was more comprehensively provided in high-income countries than in low-middle-income countries [99].

Of note, a recent survey on European nephrologists about factors influencing modality choice indicated that ~10% of patients received no information before the start of kidney replacement therapy (KRT) [98]. Early information provision and more involvement of patients in decision-making were more frequently reported in middle- and high-GDP countries (*p* < 0.05). Many respondents advocated increased uptake of home dialysis and kidney transplantation [98]. According to recent estimates, about 30% of CKD patients on KRT are late referrals, although late referral is differently defined. This implies a loss of chances for the patients, as it often occurs in the most fragile individuals, and impairs the optimization of dialysis choice [100,101,102,103].

Table 7 shows some of the most common factors associated with late referral.

In-center hemodialysis has however advantages; from the patient’s side, the desire to be assisted by competent medical and nursing staff; the awareness of having cognitive problems or difficulty in performing manual tasks; the fear of performing complex tasks and of involving family members; the desire to be in the company of people receiving the same treatment for kidney failure; the unavailability of a suitable home or the refusal to have dialysis equipment and supplies at home and, as for HHD, the difficulty or fear, of fistula puncture, especially in elderly people [104].

As previously mentioned, the focus is presently shifting from this endless controversy to maintaining the quality of life and adapting treatment to patients’ needs [105,106,107,108]. This approach emphasizes the need to consider the quality of life impairment of dialysis patients, found to be comparable to that of cancer patients [109,110].

Fatigue, chronic pain, depression, insomnia, restless legs syndrome, itching, and sexual dysfunction contribute to the quality of life impairment. Patients want to work, exercise and travel, and fear being confined in the place of care for their remaining life [106,108].

The demonization of in-center dialysis is not an answer to patients’ needs. While it is true that out-of-hospital dialysis allows better integration into daily life, in-center dialysis should be better exploited to offer patients educational sessions, and intradialytic activities, including exercise, that can improve quality of life, disease acceptance, and probably also survival [111,112].

## 9. Starting with a Full Dose or with Incremental Dialysis

The concept of incremental dialysis starts from the observation that, since the loss of kidney function is generally progressive, KRT could be progressively adapted, according to the residual renal function, until complete replacement. This concept, already expressed by Mehrotra, Nolph, and Gotch in the 90s [113], was then carried forward in the 2000s by observational studies showing that an incremental approach at the beginning of PD and, subsequently, of HD had benefits in terms of maintenance of residual renal function and was associated with better acceptance and lower initial interference with lifestyle, reducing the so-called “dialysis shock” [114,115,116]. Incremental dialysis (HD or PD) offers an individualized approach to treatment initiation; this has many potential benefits but may increase risks in patients who are not carefully selected, educated, and willing to participate in shared decision-making with their attending nephrologist [65].

In the absence of shared indications regarding incremental dialysis start, some authors suggest selecting patients according to a rather long list of requirements, usually including residual urea clearance ≥ 3 mL/min, urinary output > 0.5 L/day, good nutritional status, good electrolyte control, stable cardiovascular status and satisfactory quality of life suggesting that these parameters, and in particular residual renal function, should be checked periodically (usually monthly) [117].

Conversely, in a recent large series from France, in which over 60% of incident patients were treated by personalized, incremental, and decremental dialysis modalities, the choice was performed “in negative”, i.e., starting with personalized schedules for all of the patients without contraindications (mainly absence of rapid loss of residual kidney function and severe hypertension) [26]. In this setting, in which in-hospital patients were overall old and with a high comorbidity burden, 24 h urine collections were not employed for modulation of the treatment, whose frequency increase or decrease was defined on clinical grounds, and according to the standard laboratory data, while Beta-2 microglobulin derived formulae were used to indirectly assess residual kidney function [26,118].

Indeed, one of the debated problems regarding incremental dialysis is how to measure residual kidney function. For PD (at least for CAPD), it is intuitive that the total solute removal (PD clearance + residual renal clearance) can be directly calculated from the amount of the catabolites (e.g., urea) present in the dialysis effluent and the urines. In contrast, on HD some authors suggest using urea clearance, others prefer creatinine clearance or a mean of the two. The EBPGs [119] have suggested the calculation developed by Casino-Lopez [120], while the American NKF-KDOQI [121] suggested Gotch’s method [122]. Since 24 h urine collections are time-consuming and subject to pre-analytical errors, some authors have suggested different means for calculation, mainly based upon beta-2 microglobulin levels [118,123,124].

The advantages of an incremental dialysis start are intuitive from the patients’ side, while the results in terms of mortality are still unclear [117]. The economic advantages are obvious; the wide use of incremental dialysis also reduces the burden of in-hospital hemodialysis. Incremental dialysis is however not “low-dose” dialysis and the psychological and clinical advantages should not be offset by underdialysis [125].

## 10. Working Conclusions

The choice of dialysis modality should be adapted to the logistical situation and established in the context of a shared decision involving physicians, patients, and caregivers. Information should be as detailed as possible and cover dialysis, transplantation, and “conservative” therapy, if appropriate. While a multidisciplinary meeting is not always possible, it is highly recommended, when feasible, to involve the different professionals who take part in the usual care: nephrologists, nurses of the educational pathway, psychologists, social workers, and, if possible, expert patients. While a multidisciplinary pathway is recommended by some authors to favor an independent choice, it should be borne in mind that patients choose their treatment with their physicians and not with a structure, or with people they only meet only on one occasion. The importance of a deep and strong patient-physician relationship is well acknowledged.

**Figure 3:**
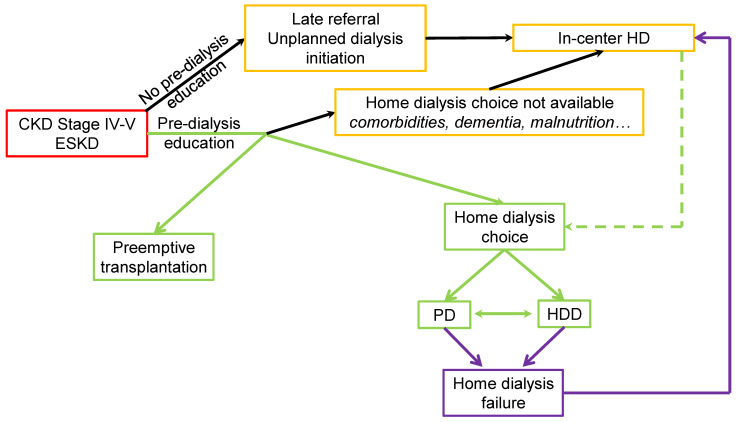
Dialysis Modality choice diagram (green lines: preferential pathways).

The choice of treatment should not be considered a one-way decision. Switching between methods should always be considered, ideally anticipating the failure of the current technique and the different dialysis modalities should be a part of an integrated system of ESKD treatment (Figure 3).

## Figures and Tables

**Table 1:** Dialysis treatments according to prescription patterns (RKF: Residual Kidney Function, IPD: Incremental Peritoneal Dialysis, IHD: Incremental Hemodialysis).

Dialysis Treatment	Description
Conventional dialysis	Three treatments per week for approximately 4 h are performed using any hemodialysis machine.
Incremental dialysis	Incremental dialysis uses the concept of adjusting dialysis doses according to RKF so that the dialysis dose is individualized. IPD was defined as <3 dwells per day in Continuous Ambulatory Peritoneal Dialysis (CAPD) and <5 sessions per week in Automated Peritoneal Dialysis (APD); IHD was defined as <3 HD sessions per week.
Intensive dialysis	More frequent and/or longer HD
Palliative dialysis	Palliative dialysis means focusing on the quality of life rather than medical parameters. Palliative care was also related to “palliative dialysis”, which is when the seriously ill patient is still on maintenance dialysis treatment, but with treatment goals being aimed at quality of life.

**Table 2:** Occurrence and overcoming of obstacles to home dialysis modalities [14,24,26].

Barriers	Strategies Overcoming Hurdles
Patient’s fear of adverse events: treatment complexity, vascular access needle injection, fear of failing, machine complication.	Educational pathway flexible, early referral, involving family members, addressed to all home treatments, risk-benefit clear knowledge, providing remote monitoring options
Patient’s psychosocial problems: lack of awareness, anxiety, interference with daily activities, financial problems, sleep disturbances, social isolation, and social considerations.	Providing appropriate resources aimed toward resolving psychological stress, interdisciplinary patient assessment, counseling, meetings with other home patients and families, addressing issues relating to the patient’s expected quality of life
Healthcare system/providers: patient position-based accessibility, the distance between patient and dialysis center, shortage of facilities, poor knowledge of the benefits and drawbacks of home dialysis	Properly emphasize the benefits of home dialysis in all educational programs, provide home training programs, providing adequate education to multidisciplinary teams about the benefits of home dialysis
Government agency: inadequate funding of home dialysis therapy	Enhancing funding and/or reimbursement for home dialysis, Improving reimbursement for training
Caregiver’s burden of responsibility: patient perceived as a burden, caregiver burnout	Early identification of supportive facilities, short-term relief care, flexibility in prescription

**Table 3:** Main theoretical advantages of home dialysis choice.

Peritoneal Dialysis (CAPD/APD)	Home Hemodialysis (HHD)
Better survival (APD = HHD)	Chance of a customized dialysis prescription (5–7/week)
More mobility and flexibility	Improved outcomes for longer and better survival
Continuous treatment can improve wellness	Performing dialysis in the comfort of your own home
Less fluid and diet restrictions	Increased sense of responsibility for self-treatment
It may be the best condition for transplantation	Flexibility in choosing a dialysis schedule
Better preservation of residual kidney function	Considerable reduction of travel to the dialysis center
Safeguarding vessels for future hemodialysis access	

**Table 4:** Contraindications to main home dialysis modalities. COPD: chronic obstructive pulmonary disease.

PD Contraindications (Absolute & Relative)	HHD Contraindications (Absolute & Relative)
inflammatory abdominal diseases	lack of vascular access
COPD	hemodynamically unstable patient
end-stage liver disease with ascites	coagulopathy
unrepaired/unrepairable hernia	
ostomies/feeding tubes	

**Table 5:** Percentage distribution of KRT modalities in incident patients [42,43,44,45,46].

Registry (Year of Reference)	HD	PD	Late Referral	Tx
USRDS (2015)	87,3	9.7	36	2.8
EDTA (2019)	86	9	---	5
UKRR(2018)	65.9	18.9	17.5	9.3
ANZDATA (2019)	71.5	19.2	17.3	---
JRDR (2018)	94.4	5.6	---	---
RIDT (2018)	87	13	---	---

**Table 6:** Main barriers contrasting correct dialysis choice (according to KDIGO recommendation).

Barriers to Dialysis Choice
Health care system
Resource availability
Reimbursement policies
Facilities availability
Dialysis center health policy/expertise
Geographic location
Patient’s social status
Absolute/ relative contraindications to a type of treatment
Patient’s health status (comorbidity, frailty, cognitive impairment…)

**Table 7:** Some of the most important factors conditioning patients’ late referral.

Reasons for Late Referral
Patients at risk of ESKD due to diabetes, hypertension, and ischemic heart disease are often referred late by their physicians, who are mainly concerned about the primary disease
Lack of awareness by general practitioners on the recognition that chronic diseases such as diabetes, hypertension, and cardiomyopathy represent a high-risk factor for CKD and ESKD
The consideration that elderly patients with chronic diseases may not survive long enough to develop ESKD
Lack of validated models that predict the progression of CKD, available for application at the patient’s bedside.
Patients’ unawareness of the complexity of their disease
Patients’ fear of dialysis
Socio-cultural inadequacy and difficulty in interacting with healthcare facilities due to logistic and/or language barriers

## Data Availability

No new data was generated for this manuscript.
